# Physiological and Pathological Transcriptional Activation of Endogenous Retroelements Assessed by RNA-Sequencing of B Lymphocytes

**DOI:** 10.3389/fmicb.2017.02489

**Published:** 2017-12-12

**Authors:** Jan Attig, George R. Young, Jonathan P. Stoye, George Kassiotis

**Affiliations:** ^1^Retroviral Immunology, The Francis Crick Institute, London, United Kingdom; ^2^Retrovirus-Host Interactions, The Francis Crick Institute, London, United Kingdom; ^3^Department of Medicine, Faculty of Medicine, Imperial College London, London, United Kingdom

**Keywords:** endogenous retroviruses, endogenous retroelements, transcription, genetic, B lymphocyte activation, B cell lymphoma, autoimmunity, cancer

## Abstract

In addition to evolutionarily-accrued sequence mutation or deletion, endogenous retroelements (EREs) in eukaryotic genomes are subject to epigenetic silencing, preventing or reducing their transcription, particularly in the germplasm. Nevertheless, transcriptional activation of EREs, including endogenous retroviruses (ERVs) and long interspersed nuclear elements (LINEs), is observed in somatic cells, variably upon cellular differentiation and frequently upon cellular transformation. ERE transcription is modulated during physiological and pathological immune cell activation, as well as in immune cell cancers. However, our understanding of the potential consequences of such modulation remains incomplete, partly due to the relative scarcity of information regarding genome-wide ERE transcriptional patterns in immune cells. Here, we describe a methodology that allows probing RNA-sequencing (RNA-seq) data for genome-wide expression of EREs in murine and human cells. Our analysis of B cells reveals that their transcriptional response during immune activation is dominated by induction of gene transcription, and that EREs respond to a much lesser extent. The transcriptional activity of the majority of EREs is either unaffected or reduced by B cell activation both in mice and humans, albeit LINEs appear considerably more responsive in the latter host. Nevertheless, a small number of highly distinct ERVs are strongly and consistently induced during B cell activation. Importantly, this pattern contrasts starkly with B cell transformation, which exhibits widespread induction of EREs, including ERVs that minimally overlap with those responsive to immune stimulation. The distinctive patterns of ERE induction suggest different underlying mechanisms and will help separate physiological from pathological expression.

## Introduction

Vertebrate genomes contain a considerable number of endogenous retroelements (EREs) with various degrees of open reading frame (ORF) integrity and replication autonomy. Occupying approximately a fifth of the mouse and human genomes, long interspersed nuclear elements (LINEs) are the largest group of EREs (Lander et al., 2001; Mouse Genome Sequencing Consortium et al., 2002). LINEs are still capable of autonomous retrotransposition in both host species. They also provide the reverse-transcriptase (RT) activity and retrotransposition machinery for mobilization of other EREs that lack long terminal repeats (LTRs), collectively known as non-LTR elements, and occasionally also of processed RNAs from cellular genes (Burns and Boeke, 2012). Distinguished by the presence of LTRs flanking the proviral genome, endogenous retroviruses (ERVs) and mammalian apparent LTR-retrotransposons (MaLRs), together comprise approximately 9.8 and 8.5% of mouse and human genomes, respectively (Lander et al., 2001; Mouse Genome Sequencing Consortium et al., 2002). ERVs may still possess and express ORFs encoding functional RT, which is necessary for the replication of non-autonomous LTR elements, such as MaLRs (Mccarthy and Mcdonald, 2004; Mager and Stoye, 2015). However, only few distinct ERVs are still replication-competent in mice (Mccarthy and Mcdonald, 2004; Mager and Stoye, 2015), and ERV replication has not been demonstrated to date in humans (Kassiotis and Stoye, 2017).

In addition to loss of replication competence as a result of sequence mutation or deletion sustained over long evolutionary periods, EREs are subject to epigenetic silencing preventing or reducing their transcription, which may otherwise produce nucleic acid and protein products with significant effects on host physiology and pathology (Kassiotis and Stoye, 2016). Epigenetic silencing of EREs is particularly potent in the germplasm, but is thought to be less effective when somatic cells alter their gene expression patterns, as part of the physiological process of their differentiation or response to stimuli or as part of the pathological process of cellular transformation (Slotkin and Martienssen, 2007). Increased ERE expression has frequently been reported as a hallmark of murine and human cancer (Kassiotis and Stoye, 2017). However, ERE induction is also characteristic of the physiological lymphocyte response to stimulation. For example, the transcriptional induction of certain groups of endogenous murine leukaemia viruses (MLVs) upon lipopolysaccharide (LPS) stimulation of murine B cells has been well documented over 3 decades ago and has been linked to B cell differentiation (Stoye and Moroni, 1983). Moreover, transcriptional induction of EREs was also described in B cells from Multiple Sclerosis (MS) patients, which were found to express elevated surface levels of ERV envelope glycoproteins (Brudek et al., 2009).

Thus, this transcriptional regulation of EREs in B cells or other hematopoietic cells may influence immune function, and both beneficial and detrimental effects have been proposed (Kassiotis and Stoye, 2016). However, what remains an open question is the degree of specificity of ERE induction during physiological or pathological conditions. Understanding the degree of overlap between those EREs that are induced as part of the normal processes of cellular activation and differentiation and those that signify cellular transformation or other pathological conditions requires detailed knowledge of ERE transcriptional patterns on a genome-wide scale, which is currently lacking.

Previous analyses of ERE expression have frequently employed PCR-based assays or microarrays, which rarely afforded element-specific or genome-wide resolution. For instance, although there are 100s or 1000s of EREs represented on commercial microarray probesets, these amount to only 0.25 and 0.04% of all genomic LTR elements and non-LTR elements, respectively (Young et al., 2014). The recent advent of RNA-sequencing (RNA-seq) techniques and the increasing availability of public RNA-seq datasets now provides the opportunity to study genome-wide ERE transcriptional regulation under a range of physiological or pathological conditions (Haase et al., 2015; Sokol et al., 2016). Here, we have analyzed ERE modulation in RNA-seq data from murine and human B cells, covering physiological B cell responses to *in vitro* and *in vivo* stimulation, as well as chronic diseases, including B cell lymphoma. Our results reveal distinct patterns of limited ERE induction during B cell cellular activation, contrasting with wide-spread ERE upregulation during B cell transformation, which indicates different underlying mechanisms.

## Materials and Methods

### Repeat Region Annotation

The precise annotation of repetitive regions is central to the accurate assessment of their activities. Until recently, this has relied upon the use of manually curated consensus sequences (Bao et al., 2015) with BLASTn-based search methods to define regions of interest. In place of these flattened representations, hidden Markov models (HMMs) can now also be used to represent repeat families, better representing the full range and variability of their sequence space (Hubley et al., 2016). This profile-based masking improves both accuracy and sensitivity, and annotates an additional 5.5 and 5.1% of the mouse and human genomes, respectively (Hubley et al., 2016). Using this method, the mouse and human genomes (GRCm38.78 and GRCh38.78, respectively) were masked using *RepeatMasker*^1^ configured with *nhmmer* (Wheeler and Eddy, 2013) in sensitive mode using the Dfam 2.0 library (v150923). *RepeatMasker* annotates LTR and internal regions separately, complicating the summation of reads spanning these divides. Tabular outputs were, therefore, parsed to merge adjacent annotations for the same element and to produce gene transfer format (GTF) files compatible with popular read-counting programs. GTF files for both genomes are freely available upon request.

### Read Mapping and Counting

The expression data used in this study have been previously described and are publicly available. Ethical review, experimental and methodological details relating to study design and data acquisition can be found in the original reports. The following accessions were used: E-MTAB-2499; GSE61608; GSE60927; GSE68769; GSE65422; GSE60424; GSE72420 and GSE62241, which are a mixture of single-end and paired-end Illumina RNA-seq reads. Adapter contamination, assessed with *FastQC*^2^, was removed using *Trimmomatic* (Bolger et al., 2014), with additional quality trimming (Q20) and subsequent length filtering (both reads of a pair ≥ 35 nts). The resulting read pairs were aligned with *HISAT2* (Kim et al., 2015) and primary mappings counted with *featureCounts* (*Subread*, Liao et al., 2014) using standard GTFs for annotated genes and the curated *RepeatMasker* GTFs for repeat regions. For accuracy and to prevent ambiguity, only reads that could be uniquely assigned to a single feature were counted. This may underestimate total expression in certain situations, but ensures confident count allocation to individual features. Features with no assigned reads across all samples within an experiment were discarded. Those remaining were normalized to account for variable sequencing depth between samples using *DESeq2* (Love et al., 2014). In comparison to the use of normalization to transcripts-per million (TPM), for example, normalized read counts do not facilitate comparison of individual feature expression levels between experiments, but are nevertheless preferable for the assessment of repetitive element expression. Methods normalizing expression to TPM or reads per kilobase million, RPKM, require the accurate knowledge of transcript lengths, which cannot simply be determined for repetitive elements and are, in fact, often variable between treatments and systems. Normalized read counts were subsequently imported into Qlucore Omics Explorer (Qlucore, Lund, Sweden) for all downstream analysis and visualization. This included all statistical comparisons, calculation of fold-changes in transcript abundance, computation of *Z*-Scores (the number of standard deviations from the mean of each variable for each data point), and plotting either *Z*-Scores or log_2_ fold-changes in heat-map form.

## Results

### ERE Modulation during Murine B Cell Activation

Induction of endogenous MLVs in LPS-stimulated B cells provided one of the earliest examples of ERE modulation upon immune cell activation (Stoye and Moroni, 1983). We therefore focused on murine B cells to examine the transcriptional response of LTR and non-LTR elements to B cell stimulation. To this end, we analyzed RNA-seq data (E-MTAB-2499) from mature B cells, isolated from the spleens of C57BL/6 (B6) mice and stimulated *in vitro* with LPS, a-IgM antibodies, or a combination of CD40 ligand (CD40L) and IL-4 (Hartweger et al., 2014).

**FIGURE 1:**
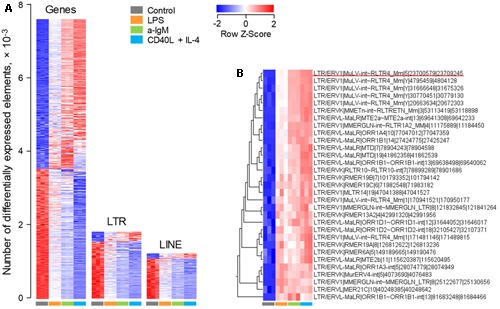
Modulation of LTR element and LINE expression upon murine B cell stimulation *in vitro*. Transcriptional analysis of purified splenic follicular B cells before and after 6-h *in vitro* stimulation with a-IgM (10 μg/ml), LPS (10 μg/ml) or a combination of CD40L (1 μg/ml) and IL-4 (0.1 μg/ml) (E-MTAB-2499). **(A)** Number of gene, LTR and LINE transcripts that are differentially expressed (≥2-fold change; *p* < 0.05) between *in vitro* activated and directly *ex vivo* isolated B cells. **(B)** The top 31 LTR EREs induced by 6-h B cell activation. In **(A,B)** each column is an independent sample. The underlined element in **(B)** is *Xmv45*.

As expected, analysis of this dataset highlighted a strong modulation of a great number of non-viral gene transcripts, with just over half of responding genes (53.6%) upregulated upon stimulation, relative to unstimulated cells (**Figure 1A**). In contrast, under the same conditions of stimulation, the majority of LTR element and LINE transcripts (85.8 and 89.3%, respectively) were proportionally downregulated (**Figure 1A**). This apparent reduction in ERE transcription is likely the effect of the increase in overall gene transcription in response to stimulation. Closer inspection of the top 31 LTR elements that were induced in these B cell upon stimulation, revealed several different groups of LTR elements (**Figure 1B**). However, notable was the over-representation of xenotropic endogenous MLVs (Supplementary Table 1). These included two closely integrated proviruses on chromosome 1, *Xmv41* (LTR/ERV1|MuLV-int∼RLTR4_Mm|1|171481146|171489815) and *Xmv43* (LTR/ERV1|MuLV-int∼RLTR4_Mm|1|170941521|170950177), which have been previously shown to be LPS-responsive (Young et al., 2014), as well as a previously unlocalized provirus, *Xmv45* on chromosome 5 (LTR/ERV1|MuLV-int∼RLTR4_Mm|5|23700579|23709245) (Supplementary Table 1). Four additional xenotropic proviruses, previously uncharacterized due to their location of the Y chromosome (Frankel et al., 1989), were also significantly upregulated (**Figure 1B** and Supplementary Table 1). However, these were highly homologous with *Xmv41, Xmv43* and *Xmv45* (Supplementary Figure 1), making it difficult to discern whether the Y-linked proviruses are genuinely expressed or whether they report expression of *Xmv41, Xmv43* or *Xmv45*.

**FIGURE 2:**
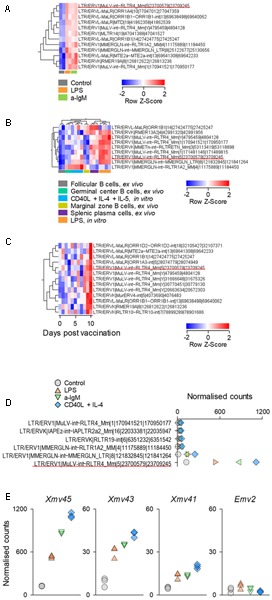
*Xmv45* induction during *in vitro* and *in vivo* murine B cell stimulation. Expression pattern of the 31 includible LTR elements identified in **Figure 1B** in three independent datasets. Significantly induced LTR elements were identified in each study separately (≥2-fold change; *p* < 0.05, and the elements shared with *in vitro* stimulated cells (**Figure 1B**) are shown. **(A)** Transcriptional analysis of purified splenic follicular B cells before and after 2-h *in vitro* stimulation with a-IgM (10 μg/ml) or LPS (25 μg/ml) (GSE61608), depicting the significantly induced LTR elements. **(B)** Transcriptional analysis of purified splenic follicular B cells before and after *in vitro* stimulation with LPS for 3 days or a combination of CD40L, IL-4 and IL-5 for 4 days (GSE60927). Also included in the comparison are *ex vivo* analyzed splenic germinal center B cells, marginal zone B cells and plasma cells. The heat map depicts the significantly induced LTR elements and unsupervised hierarchical clustering of samples according to their expression. **(C)** Mice were primed by intramuscular injection of inactivated influenza A/New Caledonia/20/99 virus and were boosted with intramuscular injection of seasonal (2006–2007) trivalent inactivated influenza vaccine 30 days later (GSE68769). The figure shows the transcriptional analysis of purified lymph node B cells, pooled from 3 mice for each of the indicated time-points after boost, depicting the significantly induced LTR elements. In **(A–C)** each column is an independent pool and the underlined element is *Xmv45*. **(D)** Normalized counts for the 6 LTR elements with the highest expression in dataset described in **Figure 1A**. Symbols represent the mean values of triplicate samples. The underlined element is *Xmv45*. **(E)** Normalized counts of the indicated proviruses in the same dataset described in **Figure 1A**. Each symbol is an independent sample.

As an independent confirmation of the observed pattern of LTR element transcriptional activation, we analyzed a second set of RNA-seq data (GSE61608) from mature B cells, isolated from spleens of B6 mice and stimulated *in vitro* with LPS or a-IgM antibodies (Fowler et al., 2015). Again, 12 out of the top 31 LTR elements identified in the previous set, were also significantly induced at the earlier time-point of 2 h in this set and, notably, these included *Xmv45* (**Figure 2A**). Furthermore, *Xmv45* was also significantly induced in a third set of RNA-seq data (GSE60927) by longer *in vitro* stimulation of B6-derived B cells with LPS or a combination of CD40L, IL-4 and IL-5 (Shi et al., 2015) (**Figure 2B**). More importantly, *Xmv45* seems also to be transcriptionally induced *in vivo*, as splenic plasma cells assessed directly *ex vivo*, which represent a state of recent B cell activation, showed elevated *Xmv45* transcription and clustered closely with *in vitro* LPS-stimulated B cells (**Figure 2B**). Lastly, further support for transcriptional induction of *Xmv45 in vivo* was provided by analysis of RNA-seq data (GSE68769) from B cells isolated from the lymph nodes of mice responding to Influenza A virus vaccination. Indeed, *Xmv45* was one of the few LTR elements that were significantly induced over the course of vaccination, despite the fact that Influenza A-specific B cells should constitute only a small fraction of total lymph node B cells that were analyzed (**Figure 2C**).

We next explored whether the consistency with which *Xmv45* transcriptional induction was detected in multiple datasets was explained by the degree of this induction. Indeed, transcription of *Xmv45* in stimulated B cells was much higher than any other induced LTR element (**Figure 2D**) and dwarfed transcription of other MLV proviruses that are either weakly (*Emv2*) or strongly (*Xmv41* and *Xmv43*) inducible by LPS stimulation (**Figure 2E**; please note that *Xmv45* expression is plotted on a scale that is 20-times higher than the rest). Together, these data suggest that transcription of a small selection of LTR elements, exemplified by *Xmv45*, is consistently induced in murine B cells by a multitude of *in vitro* and *in vivo* stimuli and validate the capacity of our analysis to detect this induction in multiple datasets.

### ERE Modulation in Murine B Cell Lymphoma

We next explored whether ERE transcriptional modulation as observed during physiological B cell activation overlapped with modulation that may occur following B cell transformation. For this purpose, we compared RNA-seq data (GSE65422) from non-transformed B cells (resting splenic B cells and germinal center B cells analyzed directly *ex vivo*; and B cells activated *in vitro* with a-CD40 and a-IgM antibodies) with B cells resembling diffuse large B cell lymphoma (DLBCL) (Zhang et al., 2015). The latter were obtained from mice that develop spontaneous B cell lymphomas as a result of deregulated expression of BCL6 under the immunoglobulin (Ig) heavy chain Iμ promoter and of deregulated activation of the alternative NF-κB pathway by expression of the NF-κB inducing kinase (NIK) under the ROSA26 promoter (Zhang et al., 2015). Both these genetic alterations were restricted to the germinal center lineage by conditional mutagenesis, using the *Cγ1-cre* transgene (Zhang et al., 2015).

**FIGURE 3:**
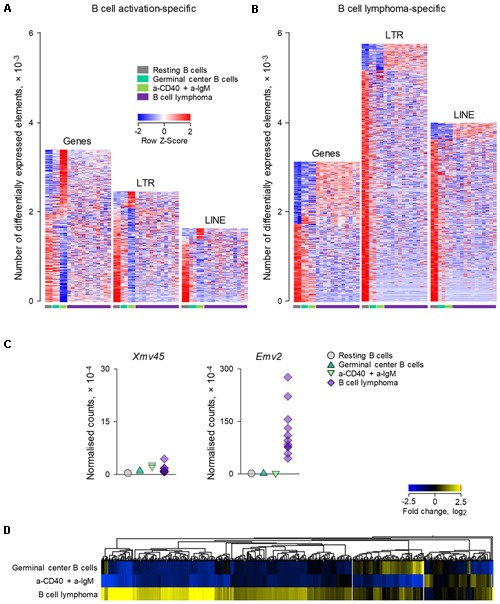
Modulation of LTR and LINE EREs following B cell transformation. **(A)** Number of gene, LTR and LINE transcripts that are differentially expressed (≥2-fold change; *p* < 0.05) between resting B cells and B cells activated *in vitro* with a-CD40 and a-IgM antibodies for 24 h (GSE65422). Also shown for comparison are germinal center B cells from wild-type mice and B cell lymphomas from mice with germinal center B cell-specific deregulation of BCL6 and NIK. **(B)** Number of gene, LTR and LINE transcripts that are differentially expressed (≥2-fold change; *p* < 0.05) in B cell lymphomas in the same dataset described above in **(A)**. Each column is an independent sample. **(C)** Normalized counts of *Xmv45* and *Emv2* transcripts in the same samples described above in **(A)**. Symbols represent individual samples. **(D)** Hierarchical clustering of LTR elements that are significantly induced in either *in vitro* activated B cells (53 transcripts) or B cell lymphomas (606 transcripts) in the same dataset as in **(A)**, in comparison with resting B cells. Mean fold changes from resting B cells are plotted.

Comparison of a-CD40 and a-IgM *in vitro* activated B cells with resting B cells in this dataset (**Figure 3A**), revealed a picture comparable with that from the previous dataset (**Figure 1A**), with transcriptional activation favoring gene induction, and to a lesser extent LTR element and LINE transcription. In contrast, LTR elements and LINEs were dominating the transcriptional differences between resting B cell and B cell lymphomas, with minimal overlap between B cell lymphomas and activated B cells (**Figure 3B**). Of note, whereas *Xmv45* was still the most induced provirus upon *in vitro* B cell activation, B cell lymphomas where characterized by significantly elevated expression of *Emv2* (**Figure 3C**). In fact, expression of *Emv2* in lymphomas was ∼50-times higher than of *Xmv45* (**Figure 3C**; please note that *Emv2* expression is plotted on a scale that is 10-times higher than that of *Xmv45*). The elevated ecotropic MLV expression likely reflects restoration of *Emv2* infectivity, which has been previously observed in cancer cell lines (Li et al., 1999) and immunodeficient animals (Young et al., 2012). In contrast to *Xmv45, Emv2* transcription in immune cells is only weakly inducible by LPS, but strongly inducible by the epigenetic derepression through BrdU treatment (Young et al., 2014), suggesting that the primary cause of its upregulation in B cell lymphomas is loss of epigenetic repression, followed by restoration of infectivity.

Consistent with different mechanistic origins of LTR element modulation during B cell activation and B cell transformation, approximately one-third of LTR elements that were transcriptionally induced in B cell lymphomas were also induced either in germinal center B cells or in *in vitro* activated B cells (in equal proportions between the two), whereas the majority (two-thirds) were unique to B cell lymphomas (**Figure 3D**).

### ERE Modulation in Human B Cells under Physiological and Pathological Conditions

Given the evolutionary divergence between EREs in different host species, we next asked whether the specificity with which EREs are modulated in murine B cells in distinct conditions, also characterized ERE modulation in B cells from a different host, namely the human. We started by investigating gene and ERE transcriptional modulation in RNA-seq data (GSE60424), generated from peripheral blood B cells, isolated from healthy individuals and those with infectious, degenerative or autoimmune diseases, including Sepsis, Amyotrophic Lateral Sclerosis (ALS), Type 1 Diabetes (T1D) and MS (Linsley et al., 2014).

**FIGURE 4:**
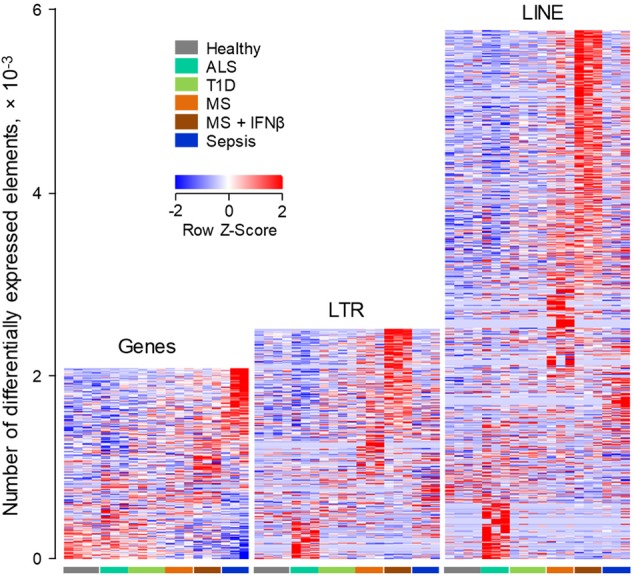
ERE modulation in human B cells from infectious, degenerative or autoimmune disease. Transcriptional analysis by RNA-seq of peripheral blood B cells isolated from healthy individuals, from patients with Sepsis, ALS or T1D and from MS patients before and 24 h after the first treatment with IFNβ (GSE60424). Heat-maps show the number of gene, LTR and LINE transcripts that are differentially expressed (*p* < 0.05) between the groups. Each column is an independent sample.

As might be expected by its acute and severe nature, Sepsis accounted for the majority of the 2,159 genes that were differentially regulated between the studied conditions, with a smaller, but clearly evident signature in MS patients shortly after the first treatment with IFNβ (**Figure 4**). A comparable number of LTR elements were also differentially expressed between the conditions (**Figure 4**). Interestingly, transcription of LTR elements appeared more distinct between the conditions, with the exception of T1D, than overall gene expression, with a particularly strong signature in the IFNβ-treated subset of MS patients (**Figure 4**). Moreover, B cells from these individuals differentially expressed more than twice the number of LINEs than of genes, with clusters of LINEs clearly distinguishing the different conditions, again with a very strong signature evident in the IFNβ-treated subset of MS patients (**Figure 4**).

**FIGURE 5:**
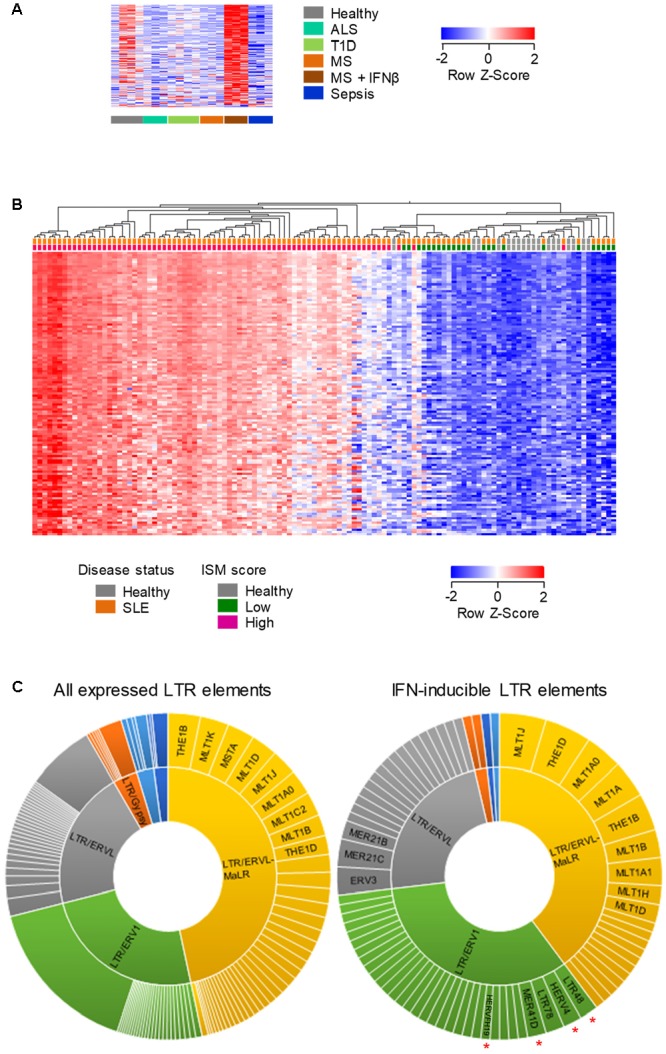
Human LTR elements induced by IFN I in whole-blood. **(A)** Expression profile of 131 LTR elements that are transcriptionally induced specifically by IFNβ treatment of MS patients, in whole-blood RNA-seq data (GSE60424). **(B)** Hierarchical clustering of healthy individuals and SLE patients according to the expression of 219 LTR elements that were induced in B cells by IFNβ treatment of MS patients and were also upregulated in RNA-seq data (GSE72420) from whole-blood samples from SLE patients as a group, compared with those from healthy individuals (≥2-fold change; *p* < 0.05). In **(A,B)** each column is an independent sample. **(C)** Diversity of the LTR elements that are expressed in peripheral blood cells (*left*) and of the 108 IFN I-inducible LTR elements that were common between purified B cells and whole-blood samples from IFNβ-treated MS patients and SLE patients. Slice widths are proportional to the frequency of each member. Significantly enriched groups (*p* < 0.05, χ^2^ with multiple comparison correction) are indicated by red asterisks.

To probe further the IFNβ-responsiveness of EREs, we first examined whether the pattern observed in purified B cells was also present when the entire complement of blood cell types was analyzed. We focused on LTR elements as they include phylogenetically more diverse groups than LINEs. Indeed, a sizeable set of the IFNβ-inducible LTR elements upregulated in B cells from IFNβ-treated MS patients was also detectable and highly induced in the same patient group, when RNA-seq data from whole-blood was analyzed (131 of 779 elements, **Figure 5A**).

We next examined the overlap between LTR elements that are induced by IFNβ treatment of MS patients and those that might be naturally induced in a setting of elevated levels of endogenously produced type I IFN (IFN I). To this end, we analyzed RNA-seq data (GSE72420) from whole-blood samples obtained from Systemic Lupus Erythematosus (SLE) patients (Hung et al., 2015), an autoimmune disease with a strong IFN I signature (Obermoser and Pascual, 2010). The intersection of LTR elements that were upregulated in purified B cells in response to IFNβ treatment of MS patients and those induced in SLE patients as a group, identified 219 common LTR elements (**Figure 5B**). Importantly, significantly elevated expression of these LTR elements was present in the vast majority (88%; 66 of 75) of SLE patients with a high Interferon Signature Metric (ISM) score, but not in any of the SLE patients with a low ISM score (0/24) or any healthy individuals (0/18) (**Figure 5B**). These results indicated that an overlapping set of LTR elements (Supplementary Table 2) were responsive to IFN I both in IFNβ-treated MS patients and in SLE patients with a high ISM score.

To explore whether IFN I was preferentially inducing certain LTR groups, we compared the composition of all LTR elements expressed in MS or SLE patients with that of the IFN I-inducible LTR elements shared between the two conditions (Supplementary Table 2). This comparison uncovered significant enrichment for the ERV1 groups as a whole, with members of the LTR48, HERV4, MER41D and HERVFH19 subgroups being frequently responsive to IFN I (**Figure 5C**). Thus, transcriptional induction of LTR elements by exogenously administered or endogenously produced IFN I displays a certain degree of specificity.

**FIGURE 6:**
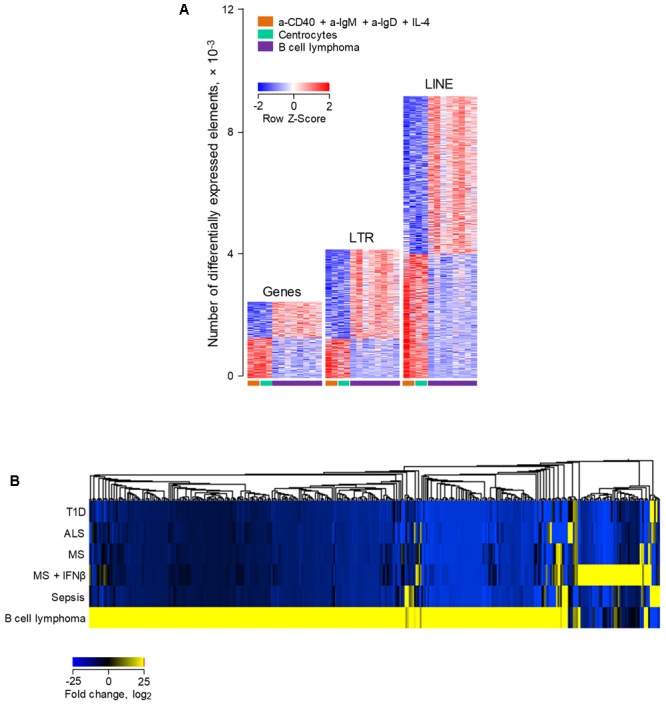
Differential modulation of LTR and LINE EREs following human B cell activation or transformation. **(A)** Number of gene, LTR and LINE transcripts that are differentially expressed (≥2-fold change; *p* < 0.01) between *ex vivo* isolated follicular lymphoma B cells and either peripheral blood B cells activated *in vitro* with IL-4, a-CD40, a-IgM, and a-IgD antibodies or *ex vivo* isolated tonsillar centrocytes (GSE62241). Each column is an independent sample. **(B)** Hierarchical clustering of a total of 395 LTR elements that are significantly induced in either e*x vivo* isolated follicular lymphoma B cells or peripheral blood B cells from infectious, degenerative or autoimmune diseases described in **Figure 4**. Mean fold changes from the respective B cell control are plotted.

Lastly, we investigated how human B cell transformation might influence ERE transcriptional behavior. To achieve this, we compared ERE transcription in RNA-seq data (GSE62241) from follicular B cell lymphoma and from non-transformed B cells (Koues et al., 2015). The groups included B cells purified from follicular lymphoma biopsies; centrocytes, the non-cycling fraction of germinal center B cells, isolated *ex vivo* from tonsillar tissues; and activated B cells, isolated from peripheral blood samples and stimulated *in vitro* using a combination of IL-4, a-CD40, a-IgM, and a-IgD (Koues et al., 2015). Consistent with their original description (Koues et al., 2015), follicular lymphoma B cells differentially expressed a substantial number of genes, in comparison with non-transformed activated B cells or centrocytes (**Figure 6A**). Notably, a much larger number of EREs were dysregulated in follicular lymphoma B cells, with twice as many LTR elements and four-times as many LINEs upregulated in follicular lymphoma B cells as genes (**Figure 6A**). More importantly, investigation of the overlap between LTR elements upregulated in follicular lymphoma B cells with those induced in B cells from diseases other than cancer uncovered distinguishable, non-overlapping patterns, with the majority of induced LTR elements specific to B cell lymphoma (**Figure 6B**). Together, these results suggested that distinct LTR elements are transcriptionally activated in cancer and in other diseases.

## Discussion

Endogenous retroelements constitute a sizeable fraction of the genome and their transcription has considerable potential to affect cellular physiology or contribute to pathology. However, their precise contribution can only be accurately assessed with detailed knowledge of their transcriptional behavior at the genome-wide level with the resolution of individual ERE integrations (Kassiotis and Stoye, 2016). The technological advances of transcriptional profiling by RNA-seq now afford a means of addressing genome-wide ERE modulation in health and disease. Using such methodology, we uncovered unique patterns of ERE modulation characteristic of physiological activation or pathological transformation of murine and human B cells.

Study of murine B cell responses to physiological innate immune, cytokine, or BCR stimuli highlighted the responsiveness predominantly of gene transcription and the lack of widespread induction of LTR elements or LINEs. In fact, most B cell-expressed EREs appeared downregulated in murine B cells upon activation, likely due to the overshadowing induction of strong gene transcription under these conditions. Prior analysis of immune cell stimulation by microarray methods had also indicated that transcription of different ERE groups could either increase or, indeed, decrease upon activation (Young et al., 2014), but lacked the resolution of RNA-seq data analysis. Our current analysis identified a small number of distinct LTR elements that are consistently activated in stimulated murine B cells, with a single endogenous MLV provirus, *Xmv45*, expressed at higher levels than all other LTR elements together. It would be interesting to explore the reasons for this unique inducibility of *Xmv45*, as well as its potential consequences for B cell function.

Contrasting the strong induction of a very limited number of ERE proviruses following B cell stimulation, expression of a much larger number of EREs was found altered following B cell transformation. Gene expression profiling of DLBCL has revealed patterns associated either with an activated B cell phenotype or with a germinal center phenotype (Lenz and Staudt, 2010). Each subtype is characterized by a different frequency of mutations in pathways affecting cellular activation, such as mutations promoting constitutive NF-κB activation, or differentiation, such as BCL6 or its antagonist Blimp-1 (Lenz and Staudt, 2010). Consistent with these observations, we found that one-third of LTR elements that were induced in B cell lymphoma cells were shared with either activated B cells or germinal center B cells. Induction of these shared LTR elements is likely to be induced by the same transcriptional regulators induced in activated B cells (e.g., NF-κB) or germinal center B cells (e.g., BCL6), which are also overexpressed in B cell lymphomas.

NF-κB, together with IRF1, have been incriminated for the transcriptional activation of HERV-K(HML2) proviruses in ALS brain tissue and human astrocytes and neurons treated with inflammatory cytokines (Manghera et al., 2016). These two immune activation-induced transcription factors are part of a longer list of nearly 40 host transcription factors that are suspected to directly drive transcription of HERVK LTRs (Manghera and Douville, 2013). Indeed, this relatively high affinity of ERV LTRs for host transcription factors seems to be an intrinsic, evolutionarily shared property (Dunn et al., 2005), and underlies their ability to establish and rewire host gene regulatory networks (Rebollo et al., 2012). Whether BCL6 or Blimp-1 directly affect transcription of ERVs is not currently known, but Blimp-1 has been reported to repress expression of HIV-1 proviruses in T cells (Kaczmarek Michaels et al., 2015). Therefore, BCL6 may induce expression of ERVs indirectly, through its established role in repressing the repressor Blimp-1 (Crotty et al., 2010).

Given the common pathways that drive B cell activation, germinal center response and B cell transformation, it was surprising to observe that the majority of LTR elements that were significantly upregulated in B cell lymphoma were unique to this condition and were not shared with physiologically activated B cells. This was exemplified by the observed expression of *Emv2*, which surpassed the highly inducible *Xmv45* by two orders of magnitude, to become the single most expressed MLV in B cell lymphoma. Whereas *Xmv45* is highly inducible by LPS, cytokine or antigenic stimulation of B cells, *Emv2* is primarily responsive to epigenetic modifiers (Young et al., 2014), implicating epigenetic changes in the altered MLV expression profile in B cell lymphoma cells. It should be noted that although expression of ecotropic MLV found in RNA-seq data is attributed here to the germline copy of *Emv2*, based on sequence identity, it may also arise from new somatically acquired integrations of an *Emv2*-derived infectious retrovirus. Indeed, the dysregulation of *Emv2* alongside the expression of complementary viruses may support the production of infectious recombinant retroviruses, further increasing observed expression, particularly in B cell lymphomas (Young et al., 2012). The same is also true for other mobile EREs, such as intracisternal A particle (IAP) elements in mice and LINEs in both humans and mice, where the reported expression is the combination of transcription of germline copies and any somatically acquired additional copies.

Akin to murine B cells, distinct patterns of LTR element and LINE expression characterized B cells isolated from patients suffering from different autoimmune, infectious, degenerative or neoplastic diseases. However, interesting differences between the two host species were also observed. Whereas the transcriptional response of murine B cells was overshadowed by gene expression changes, this was not the case in human B cells where transcription of EREs was far more responsive to the influence of the diseases studied here. This was particularly visible for LINEs, which indeed were nearly three-times more numerous than non-viral genes in the transcriptional difference between B cells from the different diseases. These findings may indicate higher overall transcriptional activity of LINEs in humans (Hancks and Kazazian, 2012).

Regardless of its origin, the enhanced transcriptional responsiveness of human EREs provides a considerably more detailed map of transcriptional activity across the genome than annotated genes alone. For example, the transcriptional response to IFN I treatment of MS patients was more evident in LTR element or LINE transcription than in gene transcription overall. The sheer number of transcribed EREs allows for increased statistical power, revealing differences that may be too subtle to detect otherwise.

Also similar to the specificity of LTR element expression in stimulated murine B cells, human B cells upregulated a select list of LTR elements in conditions of IFN I stimulation. IFN I-inducible LTR elements, shared between purified B cells and whole-blood samples and between IFNβ-treated MS patients an SLE patients, were enriched for ERV1 class elements and included LTR48, HERV4 and MER41D members. Several IFN-induced transcription factors, including NF-κB, IRFs and STATs are predicted to bind HERV-K LTRs (Manghera and Douville, 2013), indicating direct responsiveness of at least certain ERV groups to IFN stimulation. Interestingly, members of the MER41 group were recently shown to confer IFNγ-responsiveness to the *AIM2* gene and to contribute to the activation of other immune-related genes, by providing binding sites for the transcription factors IRF1 and STAT1 (Chuong et al., 2016). It is, therefore, conceivable that the IFN I responsiveness of LTR48, HERV4 and MER41D elements is mediated by IFN I-induced transcription factors.

In comparison with resting or activated B cells, the most pronounced induction of ERE transcription was witnessed in human B cell lymphoma cells, affecting thousands of LTR elements and LINEs. More importantly, as was the case with murine B cell lymphoma cells, the LTR elements that were activated in human B cell lymphoma cells exhibited minimal overlap with those expressed in non-transformed B cells from any of the conditions studied. Together, these results suggest that cellular transformation, at least in the B cell lineage, is associated with dysregulation of a non-random set of EREs that are not typically found dysregulated in other conditions. The distinctive patterns of ERE induction will help separate physiological from pathological expression, as well as provide targets for possible intervention.

## Author Contributions

GY developed the bioinformatics pipeline. JA, GY, and GK analyzed the data. JA, GY, JS, and GK wrote the manuscript. JS and GK supervised the study.

## Conflict of Interest Statement

GK is a scientific co-founder of and consulting for ERVAXX and a member of its scientific advisory board. GK, GY, and JA may receive royalties through their institution from ERVAXX. The other author declares that the research was conducted in the absence of any commercial or financial relationships that could be construed as a potential conflict of interest.

## Footnotes

1 repeatmasker.org

2 www.bioinformatics.babraham.ac.uk/projects/fastqc

## References

[B1] Bao W. Kojima K. K. Kohany O. (2015). Repbase update, a database of repetitive elements in eukaryotic genomes. *Mob. DNA* 6:11. 10.1186/s13100-015-0041-9 26045719 PMC4455052

[B2] Bolger A. M. Lohse M. Usadel B. (2014). Trimmomatic: a flexible trimmer for Illumina sequence data. *Bioinformatics* 30 2114–2120. 10.1093/bioinformatics/btu170 24695404 PMC4103590

[B3] Brudek T. Christensen T. Aagaard L. Petersen T. Hansen H. J. Moller-Larsen A. (2009). B cells and monocytes from patients with active multiple sclerosis exhibit increased surface expression of both HERV-H Env and HERV-W Env, accompanied by increased seroreactivity. *Retrovirology* 6:104. 10.1186/1742-4690-6-104 19917105 PMC2780989

[B4] Burns K. H. Boeke J. D. (2012). Human transposon tectonics. *Cell* 149 740–752. 10.1016/j.cell.2012.04.019 22579280 PMC3370394

[B5] Chuong E. B. Elde N. C. Feschotte C. (2016). Regulatory evolution of innate immunity through co-option of endogenous retroviruses. *Science* 351 1083–1087. 10.1126/science.aad5497 26941318 PMC4887275

[B6] Crotty S. Johnston R. J. Schoenberger S. P. (2010). Effectors and memories: Bcl-6 and Blimp-1 in T and B lymphocyte differentiation. *Nat. Immunol.* 11 114–120. 10.1038/ni.1837 20084069 PMC2864556

[B7] Dunn C. A. Van De Lagemaat L. N. Baillie G. J. Mager D. L. (2005). Endogenous retrovirus long terminal repeats as ready-to-use mobile promoters: the case of primate beta3GAL-T5. *Gene* 364 2–12. 10.1016/j.gene.2005.05.045 16112824

[B8] Fowler T. Garruss A. S. Ghosh A. De S. Becker K. G. Wood W. H. (2015). Divergence of transcriptional landscape occurs early in B cell activation. *Epigenetics Chromatin* 8:20. 10.1186/s13072-015-0012-x 25987903 PMC4434543

[B9] Frankel W. N. Stoye J. P. Taylor B. A. Coffin J. M. (1989). Genetic analysis of endogenous *Xenotropic murine* leukemia viruses: association with two common mouse mutations and the viral restriction locus Fv-1. *J. Virol.* 63 1763–1774. 2564439 10.1128/jvi.63.4.1763-1774.1989 PMC248440

[B10] Haase K. Mosch A. Frishman D. (2015). Differential expression analysis of human endogenous retroviruses based on ENCODE RNA-seq data. *BMC Med. Genomics* 8:71. 10.1186/s12920-015-0146-5 26530187 PMC4632268

[B11] Hancks D. C. Kazazian H. H. (2012). Active human retrotransposons: variation and disease. *Curr. Opin. Genet. Dev.* 22 191–203. 10.1016/j.gde.2012.02.006 22406018 PMC3376660

[B12] Hartweger H. Schweighoffer E. Davidson S. Peirce M. J. Wack A. Tybulewicz V. L. (2014). Themis2 is not required for B cell development, activation, and antibody responses. *J. Immunol.* 193 700–707. 10.4049/jimmunol.1400943 24907343 PMC4082722

[B13] Hubley R. Finn R. D. Clements J. Eddy S. R. Jones T. A. Bao W. (2016). The Dfam database of repetitive DNA families. *Nucleic Acids Res.* 44 D81–D89. 10.1093/nar/gkv1272 26612867 PMC4702899

[B14] Hung T. Pratt G. A. Sundararaman B. Townsend M. J. Chaivorapol C. Bhangale T. (2015). The Ro60 autoantigen binds endogenous retroelements and regulates inflammatory gene expression. *Science* 350 455–459. 10.1126/science.aac7442 26382853 PMC4691329

[B15] Kaczmarek Michaels K. Natarajan M. Euler Z. Alter G. Viglianti G. Henderson A. J. (2015). Blimp-1, an intrinsic factor that represses HIV-1 proviral transcription in memory CD4^+^ T cells. *J. Immunol.* 194 3267–3274. 10.4049/jimmunol.1402581 25710909 PMC4369419

[B16] Kassiotis G. Stoye J. P. (2016). Immune responses to endogenous retroelements: taking the bad with the good. *Nat. Rev. Immunol.* 16 207–219. 10.1038/nri.2016.27 27026073

[B17] Kassiotis G. Stoye J. P. (2017). Making a virtue of necessity: the pleiotropic role of human endogenous retroviruses in cancer. *Philos. Trans. R. Soc. Lond. B Biol. Sci.* 372:20160277 10.1098/rstb.2016.0277 28893944 PMC5597744

[B18] Kim D. Langmead B. Salzberg S. L. (2015). HISAT: a fast spliced aligner with low memory requirements. *Nat. Methods* 12 357–360. 10.1038/nmeth.3317 25751142 PMC4655817

[B19] Koues O. I. Kowalewski R. A. Chang L. W. Pyfrom S. C. Schmidt J. A. Luo H. (2015). Enhancer sequence variants and transcription-factor deregulation synergize to construct pathogenic regulatory circuits in B-cell lymphoma. *Immunity* 42 186–198. 10.1016/j.immuni.2014.12.021 25607463 PMC4302272

[B20] Lander E. S. Linton L. M. Birren B. Nusbaum C. Zody M. C. Baldwin J. (2001). Initial sequencing and analysis of the human genome. *Nature* 409 860–921. 10.1038/35057062 11237011

[B21] Lenz G. Staudt L. M. (2010). Aggressive lymphomas. *N. Engl. J. Med.* 362 1417–1429. 10.1056/NEJMra0807082 20393178 PMC7316377

[B22] Li M. Huang X. Zhu Z. Gorelik E. (1999). Sequence and insertion sites of murine melanoma-associated retrovirus. *J. Virol.* 73 9178–9186. 10516025 10.1128/jvi.73.11.9178-9186.1999 PMC112951

[B23] Liao Y. Smyth G. K. Shi W. (2014). FeatureCounts: an efficient general purpose program for assigning sequence reads to genomic features. *Bioinformatics* 30 923–930. 10.1093/bioinformatics/btt656 24227677

[B24] Linsley P. S. Speake C. Whalen E. Chaussabel D. (2014). Copy number loss of the interferon gene cluster in melanomas is linked to reduced T cell infiltrate and poor patient prognosis. *PLOS ONE* 9:e109760. 10.1371/journal.pone.0109760 25314013 PMC4196925

[B25] Love M. I. Huber W. Anders S. (2014). Moderated estimation of fold change and dispersion for RNA-seq data with DESeq2. *Genome Biol.* 15:550. 10.1186/s13059-014-0550-8 25516281 PMC4302049

[B26] Mager D. L. Stoye J. P. (2015). Mammalian endogenous retroviruses. *Microbiol. Spectr.* 3:MDNA3-0009-2014. 10.1128/microbiolspec.MDNA3-0009-2014 26104559

[B27] Manghera M. Douville R. N. (2013). Endogenous retrovirus-K promoter: a landing strip for inflammatory transcription factors? *Retrovirology* 10:16. 10.1186/1742-4690-10-16 23394165 PMC3598470

[B28] Manghera M. Ferguson-Parry J. Lin R. Douville R. N. (2016). NF-κB and IRF1 induce endogenous retrovirus K expression via interferon-stimulated response elements in its 5’ long terminal repeat. *J. Virol.* 90 9338–9349. 10.1128/JVI.01503-16 27512062 PMC5044829

[B29] Mccarthy E. M. Mcdonald J. F. (2004). Long terminal repeat retrotransposons of *Mus musculus*. *Genome Biol.* 5:R14. 10.1186/gb-2004-5-3-r14 15003117 PMC395764

[B30] Mouse Genome Sequencing Consortium Waterston R. H. Lindblad-Toh K. Birney E. Rogers J. Abril J. F. (2002). Initial sequencing and comparative analysis of the mouse genome. *Nature* 420 520–562. 10.1038/nature01262 12466850

[B31] Obermoser G. Pascual V. (2010). The interferon-alpha signature of systemic lupus erythematosus. *Lupus* 19 1012–1019. 10.1177/0961203310371161 20693194 PMC3658279

[B32] Rebollo R. Romanish M. T. Mager D. L. (2012). Transposable elements: an abundant and natural source of regulatory sequences for host genes. *Annu. Rev. Genet.* 46 21–42. 10.1146/annurev-genet-110711-155621 22905872

[B33] Shi W. Liao Y. Willis S. N. Taubenheim N. Inouye M. Tarlinton D. M. (2015). Transcriptional profiling of mouse B cell terminal differentiation defines a signature for antibody-secreting plasma cells. *Nat. Immunol.* 16 663–673. 10.1038/ni.3154 25894659

[B34] Slotkin R. K. Martienssen R. (2007). Transposable elements and the epigenetic regulation of the genome. *Nat. Rev. Genet.* 8 272–285. 10.1038/nrg2072 17363976

[B35] Sokol M. Jessen K. M. Pedersen F. S. (2016). Utility of next-generation RNA-sequencing in identifying chimeric transcription involving human endogenous retroviruses. *APMIS* 124 127–139. 10.1111/apm.12477 26818267

[B36] Stoye J. P. Moroni C. (1983). Endogenous retrovirus expression in stimulated murine lymphocytes. Identification of a new locus controlling mitogen induction of a defective virus. *J. Exp. Med.* 157 1660–1674. 10.1084/jem.157.5.1660 6189943 PMC2187009

[B37] Wheeler T. J. Eddy S. R. (2013). nhmmer: DNA homology search with profile HMMs. *Bioinformatics* 29 2487–2489. 10.1093/bioinformatics/btt403 23842809 PMC3777106

[B38] Young G. R. Eksmond U. Salcedo R. Alexopoulou L. Stoye J. P. Kassiotis G. (2012). Resurrection of endogenous retroviruses in antibody-deficient mice. *Nature* 491 774–778. 10.1038/nature11599 23103862 PMC3511586

[B39] Young G. R. Mavrommatis B. Kassiotis G. (2014). Microarray analysis reveals global modulation of endogenous retroelement transcription by microbes. *Retrovirology* 11:59. 10.1186/1742-4690-11-59 25063042 PMC4222864

[B40] Zhang B. Calado D. P. Wang Z. Frohler S. Kochert K. Qian Y. (2015). An oncogenic role for alternative NF-κB signaling in DLBCL, revealed upon deregulated BCL6 expression. *Cell Rep.* 11 715–726. 10.1016/j.celrep.2015.03.059 25921526 PMC4426003

