# Supplementary material for: Physiological and Pathological Transcriptional Activation of Endogenous Retroelements Assessed by RNA-Sequencing of B Lymphocytes
Source: Front Microbiol. 2017 Dec 12;8:2489. doi: 10.3389/fmicb.2017.02489 (PMC5733090; doi:10.3389/fmicb.2017.02489)
Supplement: Supplementary file 1 [file Table_1.PDF]

**Supplementary Table 1 | List of the top 31 LTR elements induced in murine B cells upon immune stimulation.**

| **LTR element** | **provirus** |
|---|---|
| LTR/ERV1\|MLTR14\|19\|47041388\|47041527 | |
| LTR/ERV1\|MMERGLN-int~MMERGLN_LTR\|8\|121832845\|121841264 | |
| LTR/ERV1\|MMERGLN-int~MMERGLN_LTR\|8\|25122677\|25130656 | |
| LTR/ERV1\|MMERGLN-int~RLTR1A2_MM\|4\|11175889\|11184450 | |
| LTR/ERV1\|MuLV-int~RLTR4_Mm\|1\|170941521\|170950177 | Xmv43 |
| LTR/ERV1\|MuLV-int~RLTR4_Mm\|1\|171481146\|171489815 | Xmv41 |
| LTR/ERV1\|MuLV-int~RLTR4_Mm\|5\|23700579\|23709245 | Xmv45 |
| LTR/ERV1\|MuLV-int~RLTR4_Mm\|Y\|20663634\|20672303 | |
| LTR/ERV1\|MuLV-int~RLTR4_Mm\|Y\|30770451\|30779130 | |
| LTR/ERV1\|MuLV-int~RLTR4_Mm\|Y\|31666648\|31675326 | |
| LTR/ERV1\|MuLV-int~RLTR4_Mm\|Y\|4795459\|4804128 | |
| LTR/ERVK\|MMETn-int~RLTRETN_Mm\|3\|53113419\|53118898 | |
| LTR/ERVK\|MurERV4-int\|5\|4073690\|4076483 | |
| LTR/ERVK\|RLTR10~RLTR10-int\|7\|78899289\|78901686 | |
| LTR/ERVK\|RMER13A2\|4\|42991320\|42991956 | |
| LTR/ERVK\|RMER19A\|8\|126812622\|126813236 | |
| LTR/ERVK\|RMER19B\|7\|101793352\|101794142 | |
| LTR/ERVK\|RMER19C\|6\|71982548\|71983182 | |
| LTR/ERVK\|RMER6A\|5\|149189665\|149190476 | |
| LTR/ERVL\|MER21C\|10\|40248385\|40248642 | |
| LTR/ERVL-MaLR\|MTD\|19\|41862358\|41862539 | |
| LTR/ERVL-MaLR\|MTD\|7\|78904243\|78904598 | |
| LTR/ERVL-MaLR\|MTE2a~MTE2a-int\|13\|69641308\|69642233 | |
| LTR/ERVL-MaLR\|MTE2b\|11\|115620387\|115620495 | |
| LTR/ERVL-MaLR\|ORR1A3-int\|5\|28074779\|28074949 | |
| LTR/ERVL-MaLR\|ORR1A4\|10\|77047012\|77047359 | |
| LTR/ERVL-MaLR\|ORR1B1\|14\|27424775\|27425247 | |
| LTR/ERVL-MaLR\|ORR1B1~ORR1B1-int\|13\|69638498\|69640062 | |
| LTR/ERVL-MaLR\|ORR1B1~ORR1B1-int\|13\|81683248\|81684466 | |
| LTR/ERVL-MaLR\|ORR1D1~ORR1D1-int\|12\|31644052\|31646017 | |
| LTR/ERVL-MaLR\|ORR1D2~ORR1D2-int\|18\|32105427\|32107371 | |
