# Supplementary material for: Physiological and Pathological Transcriptional Activation of Endogenous Retroelements Assessed by RNA-Sequencing of B Lymphocytes
Source: Front Microbiol. 2017 Dec 12;8:2489. doi: 10.3389/fmicb.2017.02489 (PMC5733090; doi:10.3389/fmicb.2017.02489)
Supplement: Supplementary file 2 [file Table_2.PDF]

Supplementary Table 2 | List of the 108 IFN I-inducible LTR elements, common between purified B cell and whole-blood samples from IFNβ-treated MS patients and SLE patients.

LTR element
LTR/ERV1?|LTR103_Mam|1|27666859|27667126
LTR/ERV1|HERV4_I-int~MER51C|4|88442633|88446412
LTR/ERV1|HERV4_I-int~MER51C|4|88451260|88453396
LTR/ERV1|HERVFH19-int|2|37132611|37135455
LTR/ERV1|HUERS-P3-int~LTR25|2|6838361|6847662
LTR/ERV1|LTR17|21|41422944|41423656
LTR/ERV1|LTR1D1|2|200476475|200477250
LTR/ERV1|LTR23|1|78644953|78645384
LTR/ERV1|LTR26C|4|88415752|88416311
LTR/ERV1|LTR26|1|78643359|78643964
LTR/ERV1|LTR28B|17|80317516|80318395
LTR/ERV1|LTR34|19|17412667|17413204
LTR/ERV1|LTR39|8|143031421|143031805
LTR/ERV1|LTR48|22|35660853|35661129
LTR/ERV1|LTR48|22|35661962|35662303
LTR/ERV1|LTR54B|3|146506652|146506983
LTR/ERV1|LTR56|4|168383314|168383754
LTR/ERV1|LTR6A|19|17415140|17415541
LTR/ERV1|LTR78|12|112928865|112929455
LTR/ERV1|LTR78|13|42967313|42968201
LTR/ERV1|LTR9A1|4|88440328|88440944
LTR/ERV1|MER34A|4|17578942|17579490
LTR/ERV1|MER41A|21|41422024|41422448
LTR/ERV1|MER41D|12|112988651|112988890
LTR/ERV1|MER41D|8|143016325|143016513
LTR/ERV1|MER41E|9|121219435|121219870
LTR/ERV1|MER48|12|121018155|121018523
LTR/ERV1|MER4CL34|21|41404401|41404857
LTR/ERV1|MER4D|21|41453561|41454335
LTR/ERV1|MER4E1|11|316784|317577
LTR/ERV1|MER51B|21|41461345|41461710
LTR/ERV1|MER57B1|12|112988233|112988626
LTR/ERV1|MER61C|4|88455225|88455618
LTR/ERV1|MER65D|12|121017733|121018079
LTR/ERV1|MER89|12|121019659|121019999
LTR/ERV1|PABL_B|4|88447571|88448256
LTR/ERVK|LTR22A|10|89398090|89398543
LTR/ERVL-MaLR|MLT1A-int|3|146507600|146508723
LTR/ERVL-MaLR|MLT1A0|21|41405308|41405655
LTR/ERVL-MaLR|MLT1A0|21|41446147|41446384
LTR/ERVL-MaLR|MLT1A0|21|41446721|41446850

LTR/ERVL-MaLR|MLT1A0~MLT1A0-int|4|88496848|88498563
LTR/ERVL-MaLR|MLT1A1|17|6770108|6770441
LTR/ERVL-MaLR|MLT1A1|20|57603529|57603856
LTR/ERVL-MaLR|MLT1A1|2|6731947|6732327
LTR/ERVL-MaLR|MLT1A|2|6833296|6833679
LTR/ERVL-MaLR|MLT1A|4|168357318|168357672
LTR/ERVL-MaLR|MLT1A|4|168475693|168476126
LTR/ERVL-MaLR|MLT1B|10|89399679|89400080
LTR/ERVL-MaLR|MLT1B|21|41409617|41409976
LTR/ERVL-MaLR|MLT1B|21|41447034|41447417
LTR/ERVL-MaLR|MLT1C2|2|6834056|6834483
LTR/ERVL-MaLR|MLT1C|11|5709776|5710190
LTR/ERVL-MaLR|MLT1D|2|37100272|37100432
LTR/ERVL-MaLR|MLT1D|4|168217486|168217993
LTR/ERVL-MaLR|MLT1F1|21|41381833|41382101
LTR/ERVL-MaLR|MLT1F2|1|78622488|78623056
LTR/ERVL-MaLR|MLT1H|12|112928400|112928864
LTR/ERVL-MaLR|MLT1H|4|88500135|88500630
LTR/ERVL-MaLR|MLT1I|3|146518078|146518456
LTR/ERVL-MaLR|MLT1J2|4|88411614|88412039
LTR/ERVL-MaLR|MLT1J|12|113009564|113009786
LTR/ERVL-MaLR|MLT1J|20|3687795|3688153
LTR/ERVL-MaLR|MLT1J|21|41438747|41439042
LTR/ERVL-MaLR|MLT1J|2|162269083|162269509
LTR/ERVL-MaLR|MLT1J|2|6729263|6729688
LTR/ERVL-MaLR|MLT1M|12|121026114|121026304
LTR/ERVL-MaLR|MLT1N2|12|112971074|112971446
LTR/ERVL-MaLR|MSTB1|11|57557096|57557392
LTR/ERVL-MaLR|MSTB2|2|6731662|6731900
LTR/ERVL-MaLR|THE1B-int|21|41462575|41464356
LTR/ERVL-MaLR|THE1B|21|41453000|41453366
LTR/ERVL-MaLR|THE1B~THE1B-int|21|41461951|41462267
LTR/ERVL-MaLR|THE1B~THE1B-int|2|6858460|6860364
LTR/ERVL-MaLR|THE1C~THE1C-int|1|78646414|78648605
LTR/ERVL-MaLR|THE1D|17|56885441|56885818
LTR/ERVL-MaLR|THE1D|21|41383915|41384297
LTR/ERVL-MaLR|THE1D|21|41401007|41401172
LTR/ERVL-MaLR|THE1D|2|191011563|191011889
LTR/ERVL-MaLR|THE1D|2|6855045|6855532
LTR/ERVL|ERV3-16A3_I-int|12|112972626|112972877
LTR/ERVL|ERV3-16A3_I-int|12|112974719|112975754
LTR/ERVL|ERV3-16A3_I-int|3|187376382|187379100
LTR/ERVL|LTR16A1|12|112963505|112963887
LTR/ERVL|LTR16A|12|112918875|112919317

LTR/ERVL|LTR16C|3|122556568|122556819
LTR/ERVL|LTR18B|17|42108329|42108899
LTR/ERVL|LTR62|8|143031806|143032468
LTR/ERVL|LTR67B|17|80364775|80365298
LTR/ERVL|LTR80A|1|78652380|78652616
LTR/ERVL|MER21-int|1|78666050|78667413
LTR/ERVL|MER21A|8|143016840|143017196
LTR/ERVL|MER21B|1|27669925|27670286
LTR/ERVL|MER21B|1|27670586|27670981
LTR/ERVL|MER21C|12|113011190|113011980
LTR/ERVL|MER21C|21|41449386|41450098
LTR/ERVL|MER21C|4|168460594|168461346
LTR/ERVL|MER68B|2|6885021|6885601
LTR/ERVL|MER68|2|6868769|6869353
LTR/ERVL|MLT2A1|21|41364593|41365047
LTR/ERVL|MLT2A2|2|6820404|6820793
LTR/ERVL|MLT2B1|12|112973584|112974093
LTR/ERVL|MLT2B3|1|78623489|78623954
LTR/ERVL|MLT2C1|21|41456270|41456653
LTR/ERVL|MLT2D|12|112977140|112977555
LTR/Gypsy|MamGyp-int|1|78646140|78646393
LTR/Gypsy|MamGypLTR2c|3|122531663|122532637
LTR|EUTREP16|12|56341604|56342015
