# Supplementary material for: Physiological and Pathological Transcriptional Activation of Endogenous Retroelements Assessed by RNA-Sequencing of B Lymphocytes
Source: Front Microbiol. 2017 Dec 12;8:2489. doi: 10.3389/fmicb.2017.02489 (PMC5733090; doi:10.3389/fmicb.2017.02489)
Supplement: Supplementary file 3 [file Presentation_1.PDF]

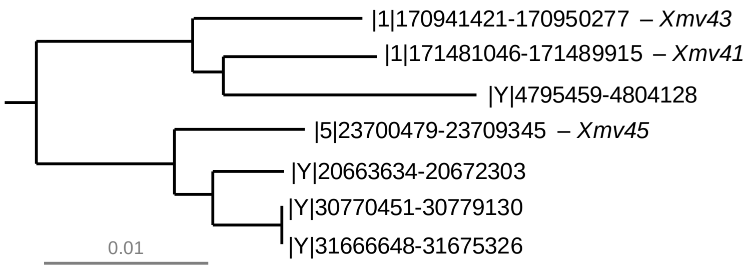

Supplementary Figure 1 | Sequence similarity between endogenous xenotropic MLVs.
Phylogenetic tree of genomic sequence homology between *Xmv41*, *Xmv43*, *Xmv45* and four endogenous xenotropic MLV proviruses on chromosome Y. Numbers denote the chromosome start and end position. The scale represents substitutions per nucleotide site.
